# Detection and Assessment of a Large and Potentially Tsunamigenic Periglacial Landslide in Barry Arm, Alaska

**DOI:** 10.1029/2020GL089800

**Published:** 2020-11-09

**Authors:** Chunli Dai, Bretwood Higman, Patrick J. Lynett, Mylène Jacquemart, Ian M. Howat, Anna K. Liljedahl, Anja Dufresne, Jeffrey T. Freymueller, Marten Geertsema, Melissa Ward Jones, Peter J. Haeussler

**Affiliations:** ^1^ Byrd Polar and Climate Research Center The Ohio State University Columbus OH USA; ^2^ Ground Truth Trekking Seldovia AK USA; ^3^ Department of Civil and Environmental Engineering University of Southern California Los Angeles CA USA; ^4^ Department of Geological Sciences and Cooperative Institute for Research in Environmental Sciences (CIRES) University of Colorado Boulder Boulder CO USA; ^5^ The Woods Hole Research Center Falmouth MA USA; ^6^ Engineering Geology and Hydrogeology RWTH‐Aachen University Aachen Germany; ^7^ Department of Earth and Environmental Sciences Michigan State University East Lansing MI USA; ^8^ British Columbia Ministry of Forests, Lands, Natural Resource Operations, and Rural Development Prince George British Columbia Canada; ^9^ U.S. Geological Survey, Alaska Science Center Anchorage AK USA

**Keywords:** landslide, tsunami, glacier retreat, satellite imagery, DEM

## Abstract

The retreat of glaciers in response to global warming has the potential to trigger landslides in glaciated regions around the globe. Landslides that enter fjords or lakes can cause tsunamis, which endanger people and infrastructure far from the landslide itself. Here we document the ongoing movement of an unstable slope (total volume of 455 × 10^6^ m^3^) in Barry Arm, a fjord in Prince William Sound, Alaska. The slope moved rapidly between 2010 and 2017, yielding a horizontal displacement of 120 m, which is highly correlated with the rapid retreat and thinning of Barry Glacier. Should the entire unstable slope collapse at once, preliminary tsunami modeling suggests a maximum runup of 300 m near the landslide, which may have devastating impacts on local communities. Our findings highlight the need for interdisciplinary studies of recently deglaciated fjords to refine our understanding of the impact of climate change on landslides and tsunamis.

## Key Points

- This study quantifies the motion of a large periglacial landslide
- It is unique that such a landslide is detected before potential failure
- This study calls attention to the glacier‐retreat‐landslide‐tsunami hazards cascade

## 1 Introduction

Global, ongoing glacier retreat is a clear manifestation of a changing climate (e.g., Shakun et al., 2015). Glaciers erode and modify glaciated mountains; they also buttress the lower slopes of mountain valleys (Deline et al., 2015; Geertsema & Chiarle, 2013; McColl, 2012; McColl & Davies, 2013). As glaciers retreat, the unsupported valley walls can deform when no longer buttressed by glacier ice (i.e., debuttressing), increasing the chance of slope instabilities (Kos et al., 2016). Landslide failure can be triggered by earthquakes (e.g., Kargel et al., 2016), prolonged or intense rainfall (e.g., Rossi et al., 2017), thawing permafrost (e.g., Darrow et al., 2016; Ward Jones et al., 2019), or snowmelt (e.g., Roberti et al., 2018). By virtue of Alaska's steep topography, high tectonic uplift rates, and high rates of temperature increase compared to lower latitude regions, the state is emerging as a hotspot for large slope failures and glaciers‐related hazards (e.g., Coe et al., 2018; Jacquemart et al., 2020).

Most landslides in glaciated regions have been studied only after failure, with sparse data available on processes preceding slope failures. Notable exceptions include Kos et al. (2016) who monitored the motion of a slow‐moving landslide using remote sensing data, quantifying the temporal and spatial relationship between landslide movements and glacier retreat. Fey et al. (2017) confirmed the response of a landslide to glacier retreat in another ice contact rockslide system. Using historical aerial photographs, Roberti et al. (2018) retrieved 60 yr of slow, precursory deformation of the large (53 × 10^6^ m^3^) 2010 Mount Meager landslide in Canada. Their investigation also revealed a direct link between slope instability and changes in the glacier below, concluding that the final collapse was triggered in summer by rapid glacier melt. In another example, Lacroix et al. (2018) measured precursory motion reaching 390 m/yr of a 3 × 10^6^ m^3^ landslide in the French Alps prior to failure.

Landslides from steep mountainsides into fjords have produced destructive tsunamis in the past (Bloom et al., 2020; Gauthier et al., 2018; Higman et al., 2018; Wiles & Calkin, 1992). For example, the 9 July 1958 landslide in Lituya Bay, Alaska (total volume of 30–60 × 10^6^ m^3^, area of 0.5 km^2^) generated a megatsunami more than 500 m high, which propagated 11 km to the mouth of the bay (Miller, 1960; Ward & Day, 2010) and caused two fatalities. Two recent large landslides near retreating glaciers include the 17 October 2015 Taan Fiord landslide in Alaska (76 × 10^6^ m^3^, 1 km^2^; Haeussler et al., 2018) and the 17 June 2017 Karrat Fjord landslide in Greenland (58 × 10^6^ m^3^, 0.5 km^2^; Gauthier et al., 2018). The Taan Fiord landslide (Dufresne et al., 2018) moved slowly for decades before suddenly failing, generating a tsunami with a runup height of 193 m on the opposite slope (Haeussler et al., 2018; Higman et al., 2018). The Karrat Fjord landslide in Greenland produced a tsunami that destroyed a large portion of Nuugaatsiaq, a town 32 km away and caused four fatalities (Gauthier et al., 2018; Poli, 2017).

**Figure 1:**
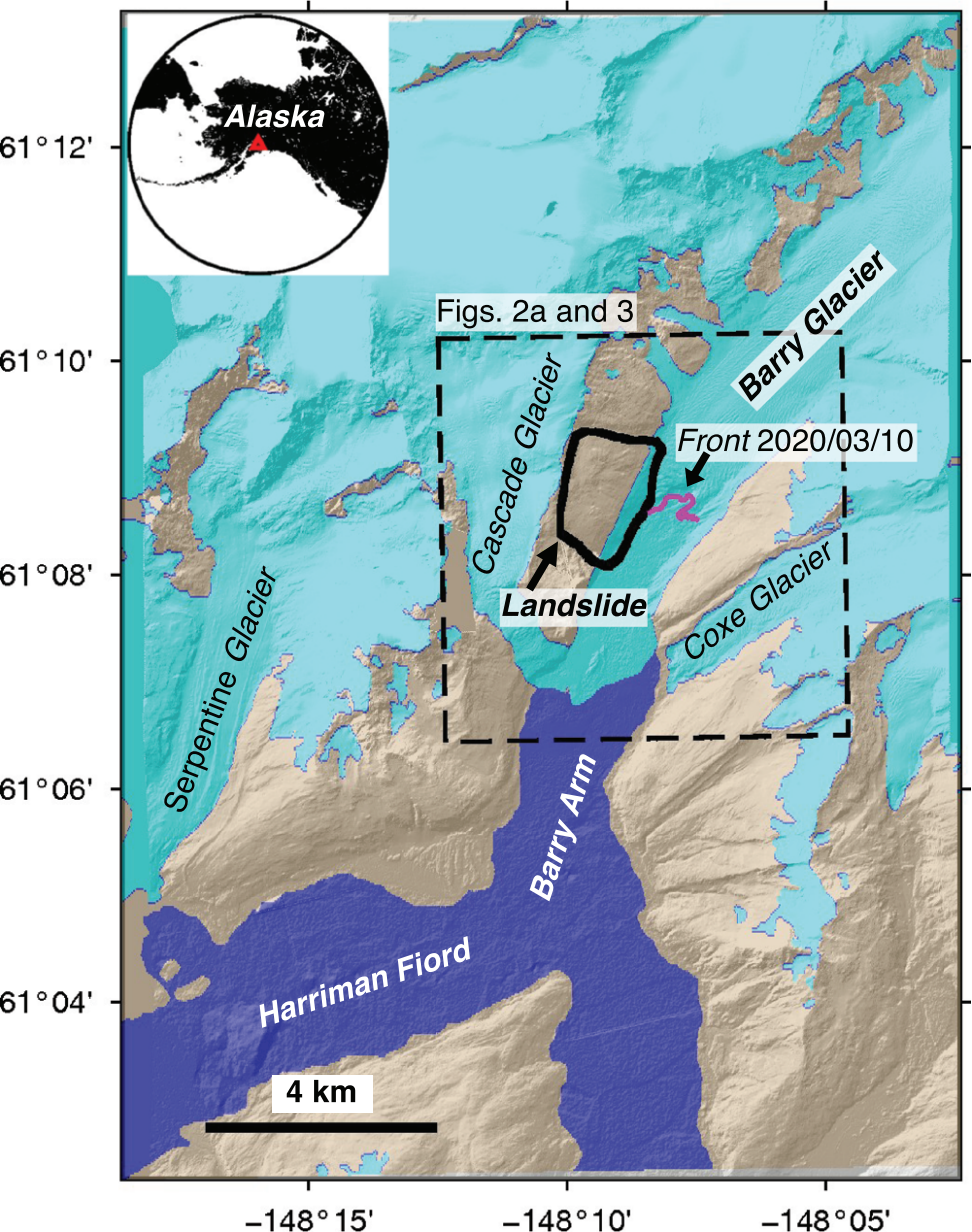
Study area and control surface. The thick black line shows the landslide outline (Figure S1b). The magenta line denotes the Barry Glacier front position on 10 March 2020 (Landsat image at 68/17, Path/Row). The gray areas were used as stable areas (i.e., control surface) for image coregistration (Text S1), the dark blue area denotes the water surface (Wessel & Smith, 1996), and the cyan area denotes the glacier coverage between 1975 and 2018 from the Randolph Glacier Inventory (RGI Consortium, 2017). Barry Glacier retreated ~3 km since the compilation of the RGI, with most of that retreat after 2006. The black box is the boundary of Figures 2a and 3. The background is the ArcticDEM hillshade. The inset image shows the location of Barry Arm landslide in Alaska. DEM(s) created by the Polar Geospatial Center from DigitalGlobe, Inc. imagery.

Here we detected, monitored, and modeled the tsunamigenic‐potential of a slow‐moving landslide above Barry Arm (herein named the Barry Arm landslide, Figure 1), a small fjord in Prince William Sound, Alaska. This landslide was discovered in the summer of 2019 by a local artist. The scarp of the landslide is visible in a 1957 aerial image but not in earlier photos (Figure S1 in the supporting information). The landslide is occurring in slate and graywacke turbidites of the Valdez Group, which is part of the Chugach accretionary complex. Much of the slide area is covered in colluvium and glacial drift; however, widespread bedrock outcrops suggest these surficial deposits account for little of the total slide volume.

## 2 Data Processing

To reconstruct the deformation history of the slope and to support estimates of potential failure, we quantified the motion of the Barry Arm landslide using digital elevation models (DEMs; 1954 to 2017), optical images (1999 to 2020), and Sentinel‐1 radar interferometry (2017–2020) (see Text S1 for details). The surface elevation changes are estimated through simple DEM differencing from a U.S. Geological Survey map DEM in 1954 (Berthier et al., 2010), SPOT5 DEM in 2006 (Berthier et al., 2010), IFSAR DEM (Interferometric Synthetic Aperture Radar) in 2010, and ArcticDEM in 2017 (Porter et al., 2018). To measure the horizontal motion of the landslide surface from repeat satellite images, we used the COSI‐Corr image correlation software of Leprince et al. (2007). We then applied an adjustment method to retrieve the cumulative horizontal displacement time series from the time series of relative horizontal displacements (Text S1). In addition, we performed an interferometric synthetic aperture radar (InSAR) analysis to assess the landslide's intra‐annual displacement patterns and recent, low magnitude deformation.

## 3 Results

### 3.1 Vertical Motions From DEM Differencing

**Figure 2:**
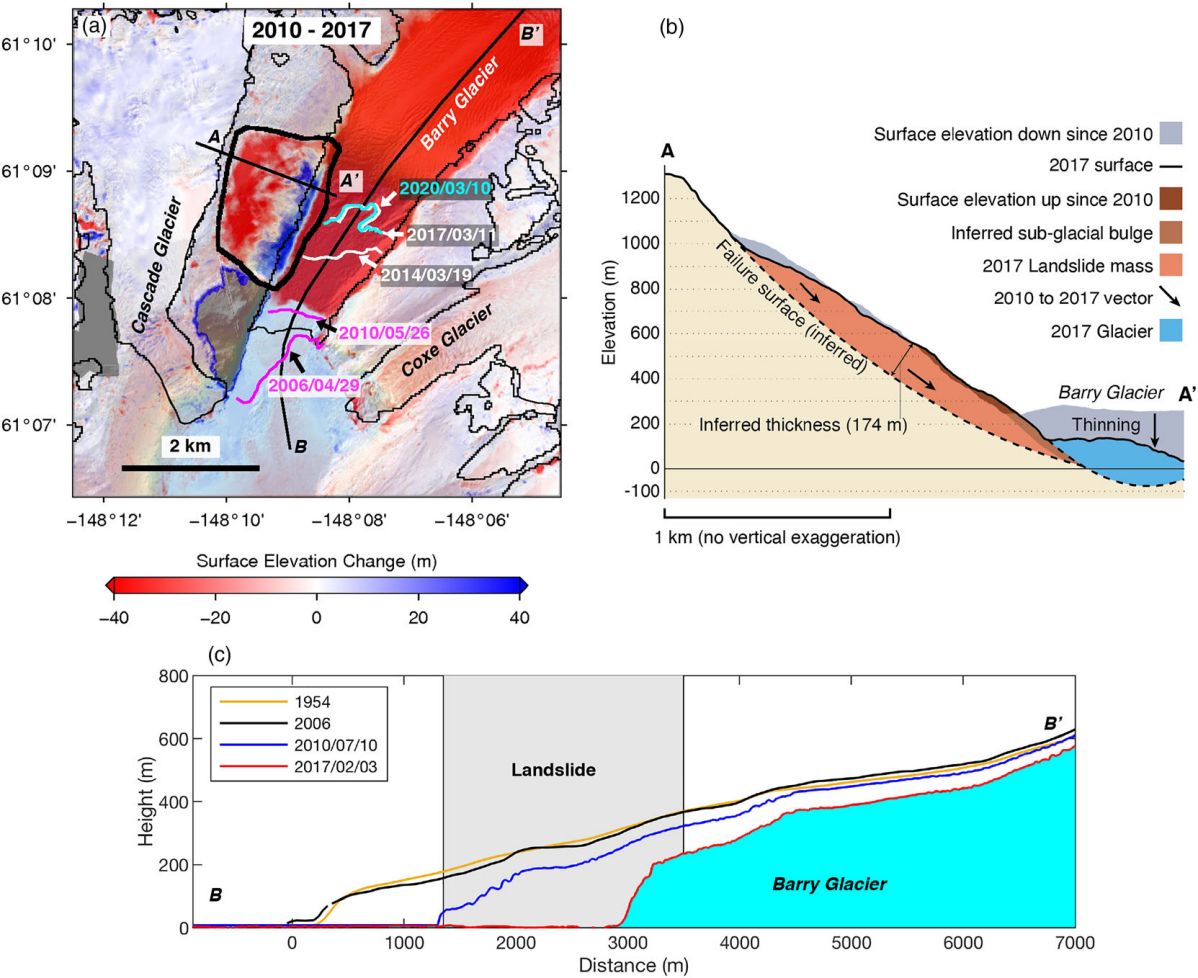
Surface elevation change and glacier retreat. (a) Surface elevation difference between 10 July 2010 (IFSAR DEM) and 3 February 2017 (ArcticDEM). The thin black lines denote the glacier outlines (2009) from Randolph Glacier Inventory. The magenta, white, and cyan lines show the Barry Glacier front positions between 2006 and 2020 from ASTER and Landsat images (Table S1). The gray areas (e.g., on the slope directly south of the Barry Arm landslide outlined by a blue line) are voids caused by blunders in the 2017 DEM. (b) The surface elevation profiles across AA′ and the inferred failure plane. (c) Surface elevation profile along Barry Glacier (BB′). Each DEM is coregistered to the 2017 ArcticDEM (referenced to the WGS84 ellipsoid). The gray shading denotes the landslide position along the glacier centerline. DEM(s) created by the Polar Geospatial Center from DigitalGlobe, Inc. imagery.

We analyzed the surface elevation changes of the Barry Arm landslide and Barry Glacier using sequential DEM differencing from 1954 to 2017 (see Text S1). During the 52 yr from 1954 to 2006, the landslide toe thickened by 30 ± 18 m (Figure S2d). Between 2010 and 2017 (Figure 2a), the surface elevation at the toe increased rapidly by 40 ± 9 m while the upper part of the slope subsided by 60 ± 9 m. The total cross‐sectional area of observed surface height increase at the landslide toe (13,000 m^2^) is smaller than the area that experienced subsidence (27,000 m^2^), because the lower part of the slope was covered by the glacier in 2010. The total surface area of the Barry Arm landslide is about 3.5 km^2^, and the total volume of the landslide block is estimated to be 455 × 10^6^ m^3^ based on the inferred geometry of the failure plane (Figure 2b; see Text S1).

**Figure 3:**
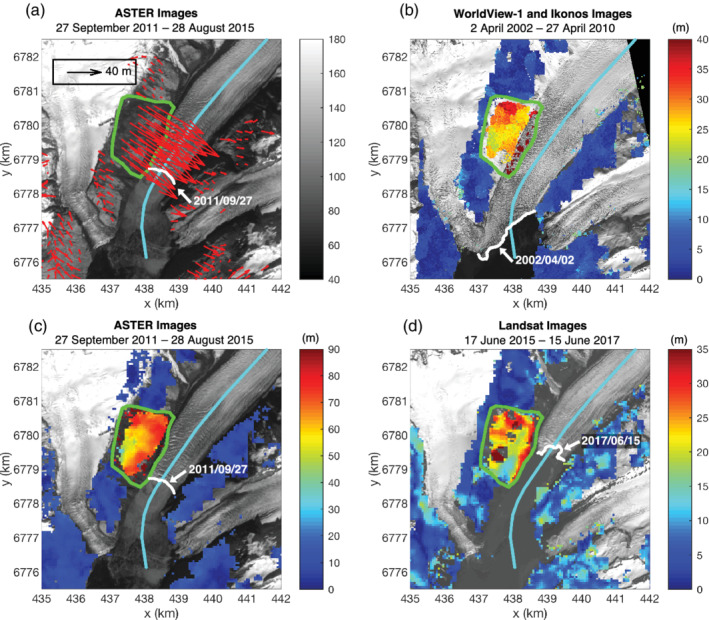
Horizontal displacement map. (a) The horizontal displacement between 27 September 2011 and 28 August 2015 from ASTER images. *x* and *y* are UTM zone 6°N coordinates. Here, the data over glaciers and water areas are masked, and the translational offsets between images have been applied. The grayscale colorbar is the digital number of the 27 September 2011 ASTER Band 2 image, and the white line is the Barry Glacier front from this image. The green line is the landslide outline. The cyan line is the Barry Glacier centerline. The red arrows represent horizontal displacement vectors. (b) The magnitude of horizontal displacement between 2 April 2002 (Ikonos image) and 27 April 2010 (WorldView‐1 image). The background is the 2 April 2002 Ikonos image, and the white line denotes the Barry Glacier front position. (c) The magnitude of displacement between ASTER images (same as panel a). (d) The magnitude of horizontal displacement between 17 June 2015 and 15 June 2017 from Landsat images. The background is the 15 June 2017 Landsat Band 8 image. *ASTER and Landsat image courtesy of the U.S. Geological Survey. Imagery © 2002–2010 DigitalGlobe, Inc.*

Barry Glacier (Figures 2c and S3) thinned by less than 1 m/yr between 1954 and 2006 (Berthier et al., 2010), but thinning increased to 40 m/yr near the terminus, after 2006. Between 2006 and 2010, the glacier retreated to the southern end of the landslide zone (~300 m/yr). Additional retreat between 2010 and 2017 brought the ice front to near the northern edge of the landslide slope. As a consequence, much of the landslide toe that terminated against the glacier margin in 2010 was exposed by 2017. The landslide toe is now at water level, as evidenced by the horizontal motion along the shoreline (Figure 3d). Total glacier thinning was 200–350 m below the southern part of the landslide toe between 2006 and 2017, while the northern portion of the glacier terminus thinned by ~100 m (Figure 2c).

### 3.2 Horizontal Motions From Satellite Imagery Pixel Tracking

We measured the horizontal displacements of the landslide with image correlation (Leprince et al., 2007) of satellite imagery from 1999 and 2020. Between 27 September 2011 and 28 August 2015, for example (Figures 3a and 3c), the median horizontal displacement over the landslide area was 56 ± 9 m for *u*_*x*_ and −36 ± 7 m for *u*_*y*_, where *u*_*x*_ and *u*_*y*_ represent the displacements along the east and north direction, respectively. The median azimuth (positive clockwise from north) of the displacement over the main scarp area is about 123°, which is consistent with the downslope direction. Figures 3b–3d demonstrate the measured displacements at different periods from Ikonos, WorldView‐1, and Landsat images. They consistently show a larger magnitude of displacement in the northern part of the landslide where the landslide is currently supported by Barry Glacier.

**Figure 4:**
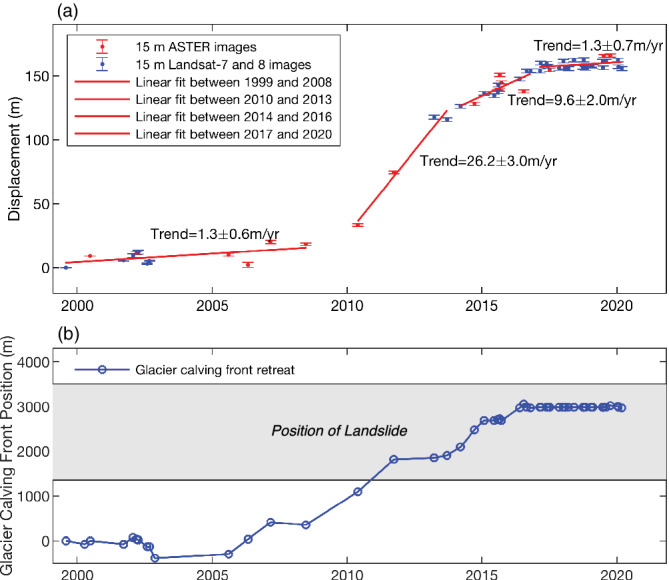
Horizontal displacement of the Barry Arm landslide and its correlation with the Barry Glacier calving front retreat. (a) The cumulative magnitude of horizontal displacement between 1999 and 2020. Red and blue error bars denote the time series of horizontal displacement (magnitude in meters) relative to the first observation (acquired on 31 July 1999). The estimated uncertainties of the displacements are about 1.3 m, which seems to be slightly underestimated because the fluctuations of the displacements are larger than the uncertainties. The red lines show the linear fit (Equation S5 in Text S1) of displacements during four periods. (b) The Barry Glacier calving front position time series. Blue circles show the glacier front positions relative to the first acquisition (31 July 1999). The gray shading denotes the landslide position along the glacier centerline.

The landslide has experienced different phases of motion (Figure 4). The relatively slow movement of 1.3 ± 0.6 m/yr from July 1999 to June 2008 was followed by a twentyfold increase, reaching 26 ± 3 m/yr, from May 2010 to September 2013. Landslide motion then slowed to 10 ± 2 m/yr from March 2014 to October 2016, and eventually returned to its pre‐2010 velocity of 1.3 ± 0.7 m/yr beginning March 2017. The recent slowdown is also supported by the analysis of high resolution (3 m) Planet imagery (Figure S4) and the InSAR analysis (Figure S8).

In order to compare the timing of the landslide displacement with glacier retreat, we measured the position of the glacier calving front in the Landsat and ASTER images following the methodology of Walsh et al. (2012). Figure 4 reveals a strong correlation (correlation coefficient = 0.99) between the position of Barry Glacier's calving front and landslide displacement. The acceleration of the landslide began in about 2010, when the terminus of Barry Glacier approached the landslide's southern edge. The landslide slowed soon after the glacier halted its retreat in 2017.

### 3.3 Deformation From InSAR Analysis

The radar interferometry analysis confirms the recent (since ~2017) slowdown of the Barry Arm landslide observed from optical imagery and reveals that displacements are currently minimal. From 2015 to 2017, displacements exceeded the threshold observable with InSAR, but pixel tracking in radar amplitude images revealed a median horizontal displacement of 19.8 m (Figure S5). During 2017, the line of sight motion (combination of horizontal and vertical motion) observed from InSAR was generally concentrated on the northeastern corner of the slide (Figure S6b), near the current glacier terminus (in agreement with pixel tracking in optical images, see Figure S4). The time series produced for the summer of 2017 suggests that the landslide may have responded to precipitation (Figure S7), but this observation is tenuous and does not seem to persist in the following years. Long‐term observations are needed before any possible links between environmental variables and landslide movement can be established. Little motion was observed in four available summer interferograms during 2018. Similarly, results from 2019 and 2020 indicate that the slide has slowed significantly (Figure S8).

### 3.4 Tsunami Simulation

**Figure 5:**
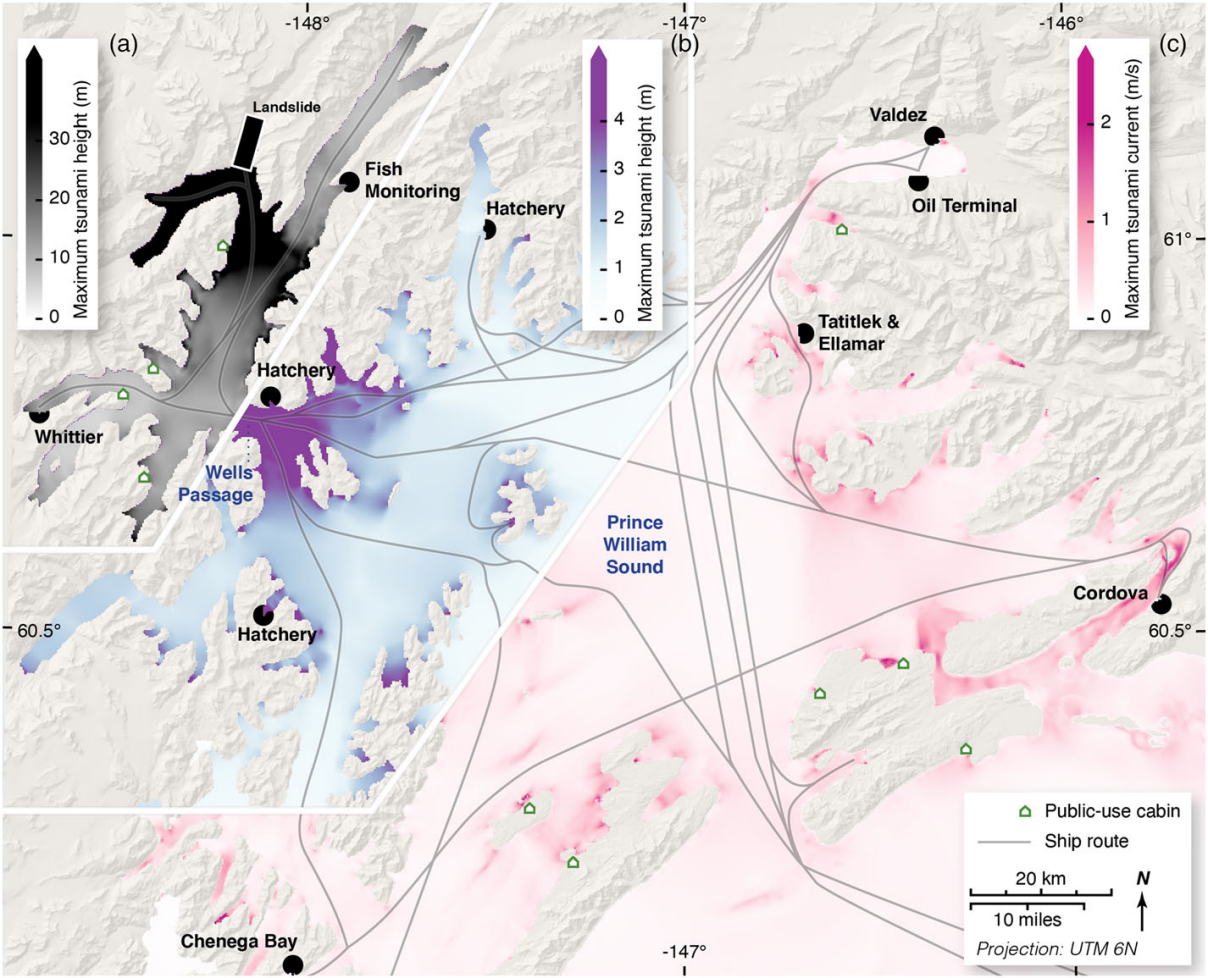
Summary of numerically predicted tsunami elevations, broken into three regions (a–c). The initial tsunami has a 150 m crest elevation near the landslide, and diminishes to tens of meters or less elsewhere in (a) including Whittier. The tsunami spreads out of Wells Passage into the rest of Prince William Sound, diminishing quickly in height, but focusing at the heads of bays (b). In southern and eastern Prince William Sound (c), the amplitude is generally under 1 m but can still generate potentially dangerous currents in shallows and passes even as far away as Cordova. Note the color scale changes for each region.

To estimate the potential tsunami hazard from a Barry Arm landslide, we use a nonlinear, dispersive (“Boussinesq‐type”) wave model (Kirby, 2016) to simulate the propagation of energy throughout Prince William Sound. We use a first‐principles approach to estimate the volume of initial tsunami. Once sufficient data are collected on the landslide, we recommend using predictive models or approaches, such as those outlined by Hungr et al. (2005) and Glastonbury and Fell (2008), to categorize the likelihood of whether the landslide will evolve into a catastrophic failure and the potential behavior and velocity of the slide mass during failures. Here, we assume that coherence and velocity of the failure are analogous to the 1958 Lituya Bay (Weiss et al., 2009) and 2015 Taan Fiord slides, and that the volume of the slide mass is much greater than the available volume of water in the channel, which yields an initial tsunami amplitude of 150 m (see Text S1 for additional details). Figure 5 and the following analysis explores what could happen if the entire landslide mass failed at once. Since the sliding masses often consist of several smaller masses that may fail sequentially (e.g., Taan Fjord landslide in 2015; Dufresne et al., 2018), other scenarios with different initial tsunami amplitudes are demonstrated in Figure S9.

Our model predicts the evolution of the tsunami crest elevation throughout Prince William Sound (Figure 5). In the immediate vicinity of the landslide, tsunami runup elevations of over 30 m are widespread, with maximum runup approaching 300 m. The maximum tsunami currents in this area are highly energetic, with speeds between 25 and 40 m/s, which would likely entrain significant bottom sediments and tsunami debris (e.g., trees). Passenger, cargo, and fishing vessels ranging from cruise ships with 5,000 people on board to individual kayaks frequent Prince William Sound and are highly vulnerable to these resulting currents. Two of the five submarine fiber optic cables into Alaska travel through outer Port Wells and may be vulnerable to turbidity currents generated by the landslide or tsunami (Piper et al., 1988). Additionally, lingering oil from the 1989 Exxon Valdez Oil Spill (Short et al., 2004) remains in unconsolidated sediment within the area of tsunami impact, presenting the possibility that tsunami erosion could remobilize this oil into the environment.

Whittier would be severely impacted by this event, with tsunami wave height in excess of 10 m arriving about 20 minutes after the slide. The tsunami wave height from this landslide scenario is similar to what was observed in Whittier during the 1964 earthquake and tsunami (Kachadoorian, 1965); however, the modeled wave period is much longer, potentially leading to more extensive inundation. Outside of Wells Passage (Figure 5), the tsunami inundation hazard is limited to a few meters above the tide, primarily at the heads of bays—presenting a hazard to campers on beaches. However, strong currents are likely throughout the Sound, and in particular at geographically constricted water channels. For example, currents near Cordova are in excess of 2 m/s, which is above the threshold for damage in marinas and harbors (Lynett et al., 2014). In areas closer to the slide, inside of Wells Passage and including Whittier, the tsunami risk could be so substantial that field monitoring and preparedness actions are necessary in order to mitigate the potentially devastating impacts of a Barry Arm landslide tsunami.

## 4 Discussion

This study identifies a large landslide that has been moving during the past 20 yr and has the potential to fail catastrophically. Our analysis reveals that the Barry Arm landslide was creeping slowly (1.3 ± 0.6 m/yr) until 2010, then accelerated (10 to 26 m/yr), before slowing down again in 2017 (1.3 ± 0.7 m/yr). The slope motion is correlated with the episode of rapid retreat (300 m/yr) and thinning (up to 40 m/yr) of Barry Glacier since 2006. Currently, Barry Glacier is only supporting one third of the 2 km wide landslide. Continued glacier retreat or thinning will expose more of the landslide toe, possibly increasing the probability of one or more catastrophic failures into the fjord.

The timing and spatial agreement between glacier retreat and slope movement suggests that the debuttressing was likely the main cause of instability between 2010 and 2017. The slow‐moving landslide accelerated as soon as the glacier retreated from the base of the landslide in 2010 (Figure 2c). The strong correlation (0.99) between glacier front position and landslide motion is consistent with Kos et al.'s (2016) finding of a direct correlation between a landslide's response and the nearby glacier retreat in Switzerland. The likelihood of continued retreat of this tidewater glacier is dependent on the bed topography, which is currently unmapped.

Results from the InSAR analysis confirm the slowdown of the landslide since 2017, and it showed that during 2017, the motion was concentrated in the northeastern corner of the landslide, closest to the glacier terminus. The downwasting of Barry Glacier likely exerted the strongest control on deformation during this time. The temporally variable deformation observed during 2017 raises questions about the current drivers of the slope instability, the answers to which are important for assessing the current hazard situation. In addition to the downwasting terminus, other environmental factors may have also affected deformation, such as precipitation, air temperature, late‐season melting of ground ice and snowmelt, and groundwater level variations. The relative contributions of these factors should be the subject of further investigations. It is noteworthy that the 2018 Anchorage earthquake (shaking intensity of about 5.3 at the landslide area; West et al., 2020) did not trigger a more rapid motion of the Barry Arm landslide.

## 5 Conclusions

We find a significant correlation between large‐scale slope destabilization in Barry Arm fjord and the retreat of Barry Glacier. As glaciers around the world retreat rapidly, exposing steep slopes prone to destabilization, the need to systematically assess the emerging hazards becomes urgent, which is now feasible with the availability of DEM time series, multiresolution satellite images and InSAR. Only by considering the entire hazard cascade and adopting interdisciplinary approaches like the one presented here can we fully understand how the hazard disposition in places like Prince William Sound may change. This understanding is critical for effective natural hazard management.

## Supporting information

Supporting Information S1

## Data Availability

The Randolph Glacier Inventory is available online (at https://www.glims.org/RGI/). Precipitation data from the Esther Island Snotel station were downloaded from https://wcc.sc.egov.usda.gov/nwcc/site?sitenum=1071 website. The shaking intensity data of the 2018 Anchorage earthquake are from the USGS Earthquake Hazards Program (https://earthquake.usgs.gov/earthquakes/eventpage/ak20419010/executive). Some figures in this paper were generated using the Generic Mapping Tools (GMT) (Wessel & Smith, 1991). The tsunami model animation generated in this work is available at https://youtu.be/AIMLqQKPhus website. Our generated data products are available at https://doi.org/10.17632/wyv78pcd59.1 website.
