# Supplementary material for: Detection and Assessment of a Large and Potentially Tsunamigenic Periglacial Landslide in Barry Arm, Alaska
Source: Geophys Res Lett. 2020 Nov 9;47(22):e2020GL089800. doi: 10.1029/2020GL089800 (PMC7816221; doi:10.1029/2020GL089800)
Supplement: Supplementary file 1 — Supporting Information S1 [file GRL-47-e2020GL089800-s001.pdf]

Geophysical Research Letters

Supplementary Information for

Detection and Assessment of a Large and Potentially Tsunamigenic Periglacial Landslide in Barry Arm, Alaska

Chunli Dai[1], Bretwood Higman[2], Patrick J. Lynett[3], Mylène Jacquemart[4], Ian Howat[1], Anna K. Liljedahl[5], Anja Dufresne[6], Jeffrey T. Freymueller[7], Marten Geertsema[8], Melissa Ward Jones[5], Peter J. Haeussler[9]

[1] Byrd Polar and Climate Research Center, The Ohio State University, Columbus, OH, USA.
[2] Ground Truth Trekking, Seldovia, AK, USA.
[3] Department of Civil and Environmental Engineering, University of Southern California, Los Angeles, CA, USA.
[4] Department of Geological Sciences & Cooperative Institute for Research in Environmental Sciences (CIRES), University of Colorado, Boulder, Colorado, USA.
[5] The Woods Hole Research Center, Falmouth, Massachusetts, USA.
[6] Engineering Geology and Hydrogeology, RWTH-Aachen University, Lochnerstr, Aachen, Germany.
[7] Department of Earth and Environmental Sciences, Michigan State University, East Lansing, MI, USA.
[8] British Columbia Ministry of Forests, Lands, Natural Resource Operations, and Rural Development, Prince George, BC, V2L 1R5, Canada.
[9] U.S. Geological Survey, Alaska Science Center, Anchorage, Alaska, USA.

Correspondence to: Chunli Dai, dai.56@osu.edu

Contents of this file

Text S1
Figures S1-S9
Table S1
Table S2

Introduction

The supporting material contains 1 text file, 2 tables, and 9 figures. The text file provides a detailed description for data processing, landslide volume estimation, and tsunami modeling. The figures support the location of the landslide from optical imagery, the surface elevation changes in the landslide area, the Barry Glacier surface elevation rate through time, horizontal displacement from Planet imagery, displacements from Sentinel-1 images, and different tsunami model scenarios. The tables include the lists of satellite images used in this study.

Text S1. Methods

DEM differencing

The surface elevation changes from 1954 to 2017 are calculated from sequential DEMs, including the 40 m resolution U.S. Geological Survey (USGS) map DEM in 1954 (*Berthier et al.,* 2010), 40 m satellite DEM (SPOT5/ASTER) in 2006 from *Berthier et al.* (2010), the 5 m resolution IFSAR (Interferometric Synthetic Aperture Radar) DEM on 10 July 2010 from USGS, and the high resolution (2 m) DEM on 3 February 2017 from ArcticDEM (www.arcticdem.org) (*Noh and Howat,* 2015, 2017; *Porter et al.,* 2018). DEM coregistration (*Nuth and Kääb,* 2011; *Noh and Howat,* 2014) is carried out to mitigate the planar offsets between pairs of DEMs. The standard deviation of elevation differences over control surfaces (gray area in Fig. 1) ranges from 9 m (the pair of the 2010 IFSAR DEM and 2017 ArcticDEM) to 18 m (the pair of the 1954 map DEM and 2006 DEM). The use of a February DEM in DEM differencing introduces additional error produced by snow presence; however, this is likely considered in the error found within the differencing of control surfaces.

Landslide slip surface and volume estimation

Characterizing landslide subsurface slip surface geometry and landslide volume can be challenging, and different methods are discussed in the literature (e.g., *Bishop,* 1999; *Aryal et al.,* 2015). Here, we infer the failure surface by fitting the surface slip vectors and constraints on the head and toe positions of the landslide. Referring to Fig. 2(b), we separate the 2010 DEM profile into upper and middle landslide segments, and translate these segments until they best match the corresponding sections of the 2017 DEM profile. This produced two 2D motion vectors that are broadly consistent with horizontal motion vectors derived from satellite imagery analysis. By assuming these vectors are (1) parallel to the basal failure surface, (2) the exposed scarp at the head of the slide is an extension of the failure surface, and (3) the failure surface is roughly circular in cross section, we estimated the shape of the upper 2/3 of the failure plane. We have little constraint on the lower portion of the failure surface, except that it likely does intersect the surface since the slide has moved several hundred meters and only the free surface can accommodate such motion. Farther south, we suspect the failure intersects the surface below the water line, since the waterline appears to be translating between successive satellite images. To complete our initial estimate of the location of the slide plane, we simply assumed that the failure surface daylights near sea level. This implies a slight tightening of the curve toward the base of the landslide.

To check whether the inferred failure surface is reasonable, we also estimate the depth of the landslide failure surface based on the balanced cross-section method (for translational landslides) (*Bishop,* 1999). Although our slip surface indicates a rotational landslide, we suspect that errors caused by the violation of model assumption are small (*Aryal et al.,* 2015). The balanced cross-section method assumes that the missing area of the mass in the depletion zone is balanced by the amount of mass flow at the translational part of the slip. Therefore, the depth of the landslide slip plane can be estimated by:

$d=A/X$

where, d is the estimated depth of the slip plane, A is the total area (in the cross section) of the depletion zone, and X is the magnitude of the displacement along the downslope direction.

For the AA' profile in Fig. 2(b), the cross-sectional area loss in the depletion zone between 2010 and 2017 is about 27,000 m^2. The downslope displacement is estimated as 155 m by matching

DEM profiles between 2010 and 2017, which agrees with the inferred downslope displacement, 145 m (120m/cos(34º)), projected from the horizontal displacement (120 m, Fig. 4(a)) between 2010 and 2017. The above equation yields a depth of slip surface as 174 m (Fig. 2(b)). This estimated thickness was very close to the thickness inferred from our circular failure surface. We made a small adjustment so that they were exactly matched.

The balanced cross-section method could also be used to constrain the slip surface below the ice surface in 2010. The cross-sectional area of increase at the bottom is only 13,000 m^2, so over half the elevation increase at the bottom would be under the 2010 ice level. The slip surface can then be fitted based on the depth of slip surface, daylighting and headwall scarp location.

The total volume of landslide mass is roughly estimated based on a single transect of the slide geometry (Fig. 2(b)). Along the AA' profile, the inferred slip surface has an average depth of 130 m. Applying that thickness to the total landslide area (on the surface) (about 3.5 km^2) yields a total volume of 455 million m^3.

Optical satellite imagery preprocessing and image correlation

Horizontal displacements over the landslide area are retrieved from optical satellite images between 1999 and 2020, including 15 m resolution Landsat 7 (1999 to present) and Landsat 8 (2013 to present) Level 1 panchromatic (Band 8) images, 15 m resolution ASTER (1999 to present) Level 1 images (Band 2, wavelength 0.63 - 0.69 m) (product: precision terrain corrected registered at-sensor radiance (AST_L1T)), 0.32 – 1.03 m panchromatic-band satellite imagery acquired by DigitalGlobe satellites such as Ikonos, GeoEye-1, QuickBird-2, WordView-1, 2 and 3, as well as 3 m resolution Planet imagery. We manually selected the images that have no cloud cover in the study area (Table S1) to retrieve the relative displacements between each pair of images. It is noted that the Landsat 7's scan-line corrector failed on May 31, 2003, which makes it difficult to retrieve relative displacements, causing data gaps between 2003 and 2013 from Landsat imagery. The gaps are filled with ASTER imagery.

Precise coregistration is the critical step to retrieve bias-free displacement estimates from imagery (*Leprince et al.,* 2007). The relative displacement between a pair of images may contain systematic offsets, so we use the average displacement over control points, where the terrain surface is stable (e.g., rock areas), to remove any translational offsets. Large areas of fast-changing surfaces such as glaciers and water areas in our study area can be identified using the glacier masks from the Randolph Glacier Inventory (RGI Consortium, 2017) and the water mask from the Global Self-consistent, Hierarchical, High-resolution Shoreline Database (GSHHS) provided by *Wessel and Smith* (1996). We also map the outline of the landslide area based on one of the Landsat 8 images (acquired on 17 June 2015). Excluding the glaciers, water, and landslide area, we can obtain a control surface (e.g., gray area in Fig. 1). Taking a pair of ASTER images between 27 September 2011 and 28 August 2015 as an example (Fig. 3 (c)), within the control surface, those displacements that have irregular directions are filtered out, i.e., the standard deviation of displacement directions within the surrounding nine pixels is larger than 60º. In addition, we filter out outliers that deviate from the median value over 3 times the standard deviation. The above two criteria yield a selection of control points, e.g., around 3100 pixels with 60 m pixel size in Fig .3(c). The average offsets (median value) between two images can be calculated from displacements over control points. Then, the offsets can be applied to the displacement map to remove the

systematic offsets (i.e., image coregistration). In this example, the systematic offsets over control points are - 40 m and 78 m along the x (east) and y (north) directions, respectively. The standard deviations of the displacement over control points are 9 m (east) and 7 m (north). Finally, we use the median value of displacements within the landslide area to represent the relative displacement between two acquisitions. Within the scarp, those displacement vectors that are more than 20º apart from the median direction are filtered out.

Least-squares adjustment to retrieve time series of displacements

For N images, there are N(N-1)/2 pairs of relative displacements from COSI-Corr. Here we developed a new algorithm to retrieve the cumulative displacement time series by taking advantage of the repeat estimates of relative displacements from all possible pairs of images. For example, given four images (A, B, C, D), there will be 6 pairs of relative displacements, which are related to each acquisition's cumulative displacement as:

$u_{A,B} = u_A - u_B$

$u_{A,C} = u_A - u_C$

$u_{A,D} = u_A - u_D$

$u_{B,C} = u_B - u_C$

$u_{B,D} = u_B - u_D$

$u_{C,D} = u_C - u_D$

where u_A, u_B, u_C, u_D are cumulative displacements at each acquisition.

The observation equation can be written in a matrix form as:

$$y = A\xi + e \qquad (1)$$

where, y is the n by 1 vector of relative displacements, e.g.,

$y = [u_{A,B}\ u_{A,C}\ u_{A,D}\ u_{B,C}\ u_{B,D}\ u_{C,D}\ ]^T$, A is the design matrix,

$$A = \begin{bmatrix} 1 & -1 & 0 & 0 \\ 1 & 0 & -1 & 0 \\ 1 & 0 & 0 & -1 \\ 0 & 1 & -1 & 0 \\ 0 & 1 & 0 & -1 \\ 0 & 0 & 1 & -1 \end{bmatrix},$$

ξ is the m by 1 vector of parameters to be estimated, e.g., $\xi = [u_A\ u_B\ u_C\ u_D\ ]^T$, and e is the n by 1 vector of errors. The dispersion of e is $\Sigma = \sigma_0^2 P^{-1}$, where σ_0^2 is the reference variance and P is the weight matrix. The dispersion matrix can be constructed from the uncertainties estimated from the coregistration of each pair of images (e.g., 10 m for ASTER image pairs).

Notice the above observation equation (equation (1)) is a rank-deficient linear system. Here we pick the first observation as the reference, so we have a fixed constraint $u_A = 0$. Its matrix form is:

$$k_0 = K\xi \qquad (2)$$

where K is a 1 by m vector, e.g., $K = [1\ 0\ 0\ 0\ ]$, k_0 is the fixed constraint, k_0=0.

The least-squares solution of the above linear system with the fixed constraint is:

$$\hat{\xi} = (N + K^TK)^{-1}c + (N + K^TK)^{-1}K^T[K(N + K^TK)^{-1}K^T]^{-1}[k_0 - K(N + K^TK)^{-1}c]$$

where, $N = A^TPA,$ and $c = A^TPy$.

The estimated reference variance can be calculated as

$$\hat{\sigma}_0^2 = \frac{\tilde{e}^TP\tilde{e}}{n-m+l} \qquad (3)$$

where $\tilde{e}$ is the estimated error, $\tilde{e} = y - A\hat{\xi}$, and l is the rank of the constraint equation (2), which is 1 here.
Hence, the dispersion matrix of the estimated parameters $\hat{\xi}$ can be calculated as
$$D(\hat{\xi}) = \hat{\sigma}_0^2(N + K^T K)^{-1} - \hat{\sigma}_0^2(N + K^T K)^{-1}K^T[K(N + K^T K)^{-1}K^T]^{-1}K(N + K^T K)^{-1} \quad (4).$$

The above equations (1 to 4) are applied to the displacements along the east (u_x) and north (u_y) direction separately. Here, x and y are coordinates in the Universal Transverse Mercator (UTM) zone 6N coordinate system. There are 153 pairs of relative displacements from 18 individual ASTER images, and 703 pairs of relative displacements from 38 individual Landsat 7 and 8 images. The cumulative displacement (u_x, u_y) time series are calculated for these two sets of data. There are small biases between them since the first measurement of each data set is used as the reference, i.e., a bias of 9 m for u_x and a bias of -0.03 m for u_y are applied to the ASTER displacement time series in order to align it with the Landsat displacement time series. The magnitude of the displacement is calculated as $u_s = \sqrt{{u_x}^2 + {u_y}^2}$. The uncertainty of the displacement magnitude is about 1.4 m for Landsat images and about 1.2 m for ASTER images, which are calculated from the error propagation of the uncertainty of u_x and u_y (obtained from equation (4)).

A linear trend model can be used to fit the displacement time series. We adopt a simple model as
$$y = a + bt \quad (5)$$
where a is the constant, in meters, b is the displacement rate, in meters/year. Based on the standard least-squares adjustment, the rate of motion can be estimated, as well as its uncertainty through error propagation.

Interferometric Synthetic Aperture Radar (InSAR) data processing
To assess the intra-annual changes of the Barry Arm landslide, we use data from Sentinel-1A and 1B (ascending orbit, track 65, frame 194). We downloaded Single-Look-Complex (SLC) images from the Alaska Satellite Facility (ASF) Vertex portal. To minimize effects from snow, we only worked with images acquired between 1 May and 31 October. To be able to remove the topographic phase from the wrapped interferograms, we downloaded the 5m Alaska IFSAR DEM from the USGS EarthExplorer platform (https://earthexplorer.usgs.gov/). Interferograms were formed using JPL open-source InSAR Scientific Computing Environment (ISCE), and initially multi-looked images with 3 azimuths and 7 range looks. Interferograms that yielded promising results were individually processed at higher resolution, using 1 azimuth and 3 range looks to yield approximately 15m x 15m pixels. Phase unwrapping was performed using Statistical-Cost, Network-Flow Algorithm for Phase Unwrapping (snaphu) method (*Chen & Zebker,* 2000). Unwrapped phase can subsequently be converted to deformation as:

$$d = \frac{\varphi\lambda}{4\pi},$$

where d is deformation in m, $\varphi$is the unwrapped phase in radians, and λ is the radar wavelength in m. We did not perform any atmospheric corrections. Lastly, we selected a reference region on a (presumably) stable part of the slope south of the slide (black star in Fig. S6) and subtracted the average deformation around the reference point (~200 m radius) from the deformation image.

Tsunami modeling

In our modeling, we use the first-principles approach to develop an initial tsunami condition; we do not employ any detailed or dynamic modeling (e.g., *Grilli et al,* 2019) to generate waves from a landslide realization. Here, we assume that the slide fails coherently and quickly, similar to the inferred tsunamigenic failures of the 1958 Lituya Bay and 2015 Taan Fiord events. More precisely, we suppose a slide that remains as a single large mass upon entering the water, and moves at a speed greater than the long wave celerity such that it evacuates all of the water in upper Barry Arm over an area equal to the initial surface area of the slide mass. The mid-channel depth in Barry Arm is assumed to be 150 m (approximated value measured by depth finders on boats), and this is the peak amplitude of the initial tsunami. The cross-channel profile of the initial tsunami follows a Gaussian shape, with a half width of 1 km. In the along-channel direction, the tsunami is described as a half-sine wave with length 2 km, yielding a displaced volume of water equal to 1/3 the volume of the landslide. The initial speed under this wave is calculated with nonlinear long wave theory with a channel-aligned direction pointing away from the landslide source. This approach is similar in concept to that found in *Borrero et al.* (2020) for the waves generated by the 2018 Anak Krakatau flank collapse. We reiterate that this solution is used to provide the order of magnitude estimates for the generated crest elevation and horizontal length scales and is not meant to capture the complex source dynamics of a potential Barry Arm failure. However, we believe it is a reasonable approximation for a case such as Barry Arm, where the landslide volume is much greater than the volume of water in the channel. Four different tsunami scenarios with various initial conditions (initial crest elevations of 25, 75, 150, and 300 m) are also carried out and demonstrated in Fig. S9.

The initial tsunami waveform is placed as a hot-start initial condition in the pCOULWAVE hydrodynamic model (*Lynett,* 2006), which provides the evolution of the landslide-generated tsunami throughout Prince William Sound. The pCOULWAVE model solves the Boussinesq-type water wave equations, including the effects of strong nonlinearity and frequency dispersion. Inclusion of dispersion can be important with landslide-generated tsunamis, which tend to have shorter wave periods than tsunamis from great subduction zone earthquakes. A single pCOULWAVE model grid, with resolution of 200 m, covers the Prince William Sound; nested grids were not used in these simulations. Bottom friction is approximated with Manning's formulation and a spatially constant "n" value of 0.025 $m^{1/3}$.

Disclaimer

Any use of trade, firm, or product names is for descriptive purposes only and does not imply endorsement by the U.S. Government.

References

Aryal, A., Brooks, B.A. and Reid, M.E., 2015. Landslide subsurface slip geometry inferred from 3-D surface displacement fields. *Geophysical Research Letters,* 42(5), pp.1411-1417.

Berthier, E., Schiefer, E., Clarke, G.K., Menounos, B. and Rémy, F., 2010. Contribution of Alaskan glaciers to sea-level rise derived from satellite imagery. *Nature Geoscience*, *3*(2), pp.92-95.

Bishop, K.M., 1999. Determination of translational landslide slip surface depth using balanced cross sections. *Environmental & Engineering Geoscience*, (2), pp.147-156.

Borrero, J., Solihuddin, T., Fritz, H., Lynett, P., et al. (20 co-authors), 2020. Field survey and numerical modelling of the December 22, 2018 Anak Krakatau tsunami. *Pure and Applied Geophysics,* doi:10.1007/s00024-020-02515-y, 2020.

Chen, C.W. and Zebker, H.A., 2000. Network approaches to two-dimensional phase unwrapping: intractability and two new algorithms. *JOSA A*, *17*(3), pp.401-414.

Grilli, S.T., Tappin, D.R., Carey, S., Watt, S.F., Ward, S.N., Grilli, A.R., Engwell, S.L., Zhang, C., Kirby, J.T., Schambach, L. and Muin, M., 2019. Modelling of the tsunami from the December 22, 2018 lateral collapse of Anak Krakatau volcano in the Sunda Straits, Indonesia. *Scientific Reports*, *9*(1), pp.1-13.

Leprince, S., Ayoub, F., Klinger, Y. and Avouac, J.P., 2007, July. Co-registration of optically sensed images and correlation (COSI-Corr): An operational methodology for ground deformation measurements. In *2007 IEEE International Geoscience and Remote Sensing Symposium* (pp. 1943-1946). IEEE.

Lynett, P.J., 2006. Nearshore wave modeling with high-order Boussinesq-type equations. *Journal of Waterway, Port, Coastal, and Ocean Engineering*, *132*(5), pp.348-357.

Noh, M.J. and Howat, I.M., 2014. Automated coregistration of repeat digital elevation models for surface elevation change measurement using geometric constraints. *IEEE Transactions on Geoscience and Remote Sensing*, *52*(4), pp.2247-2260.

Noh, M.J. and Howat, I.M., 2015. Automated stereo-photogrammetric DEM generation at high latitudes: Surface Extraction with TIN-based Search-space Minimization (SETSM) validation and demonstration over glaciated regions. *GIScience & Remote Sensing*, 52(2), pp.198-217.

Noh, M.J. and Howat, I.M., 2017. The surface extraction from TIN based search-space minimization (SETSM) algorithm. *ISPRS Journal of Photogrammetry and Remote Sensing*, 129, pp.55-76.

Nuth, C. and Kääb, A., 2011. Co-registration and bias corrections of satellite elevation data sets for quantifying glacier thickness change. *The Cryosphere*, *5*(1), pp.271-290.

Porter, C., Morin, P., Howat, I., Noh, M.J., Bates, B., Peterman, K., Keesey, S., Schlenk, M., Gardiner, J., Tomko, K., Willis, M. et al., 2018. ArcticDEM. *Harvard Dataverse*, V1, https://doi.org/10.7910/DVN/OHHUKH [Accessed May 15, 2020].

RGI Consortium (2017). Randolph Glacier Inventory – A Dataset of Global Glacier Outlines: Version 6.0: Technical Report, Global Land Ice Measurements from Space, Colorado, USA. Digital Media. DOI: https://doi.org/10.7265/N5-RGI-60

Wessel, P. and Smith, W.H., 1996. A global, self-consistent, hierarchical, high-resolution shoreline database. *Journal of Geophysical Research: Solid Earth*, 101(B4), pp.8741-8743.

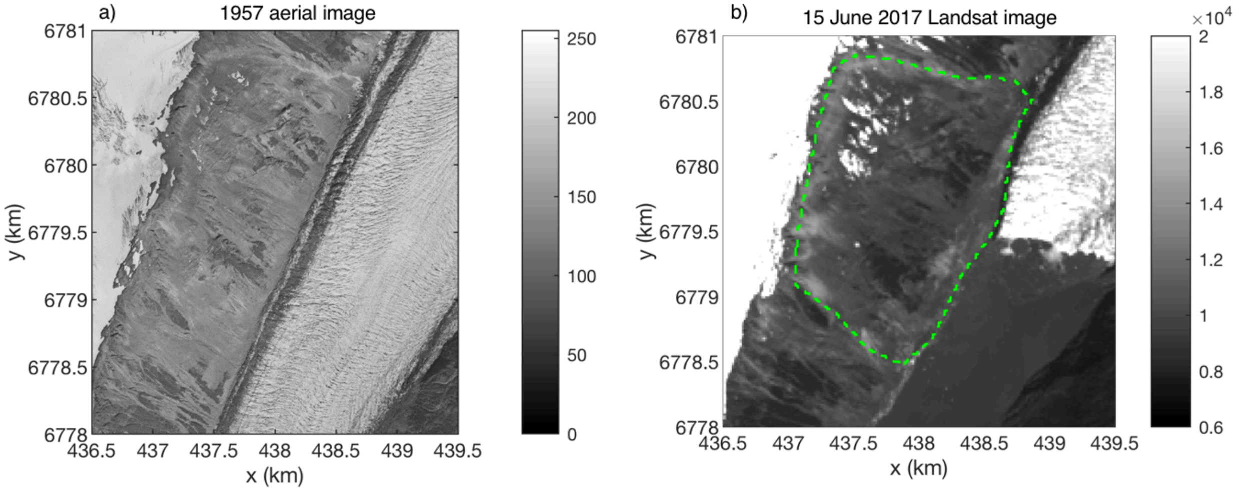

Fig. S1. Barry Arm landslide outline. (a) a 1957 aerial image from USGS. The landslide scarp is visible in the image. (b) a Landsat image acquired on 15 June 2017. The green dash line is the landslide outline manually drawn from this image (b). *x* and *y* are UTM zone 6N coordinates. The color bars represent the gray scale values of images.

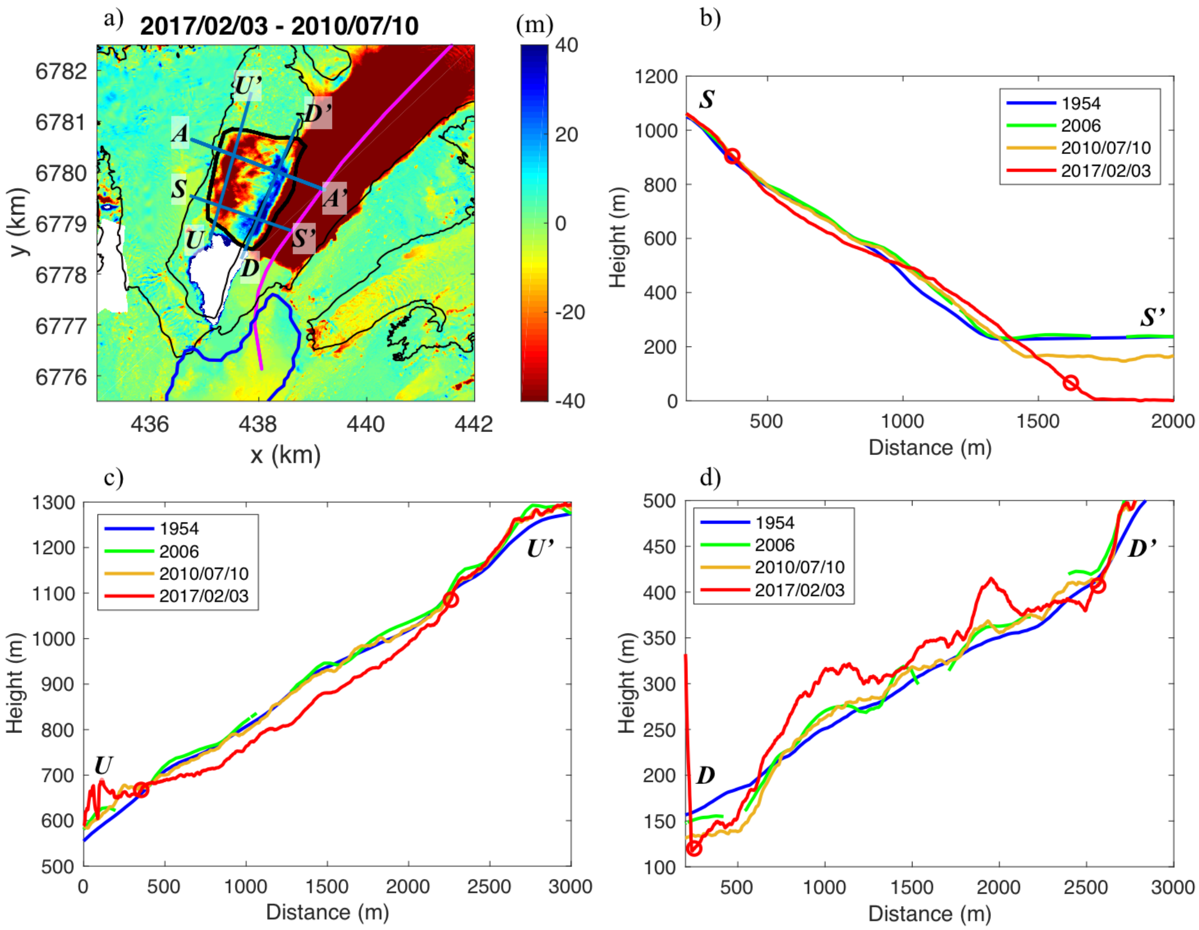

Fig. S2. Surface elevation variation across the landslide area. (a) DEM difference between February 3, 2017 and July 20, 2010. This figure is the same as Fig. 2(a) except that more cross sections are marked. The blue line is the Barry Arm fjord shoreline from GSHHS database, the thin black lines are the glacier outlines from Randolph Glacier Inventory (data source on July 16, 2007). (b)-(d) The surface elevation profiles along SS', UU', DD', respectively. The markings are the same as in Fig. 2. The red circles mark the intersections of profiles and the landslide outline.

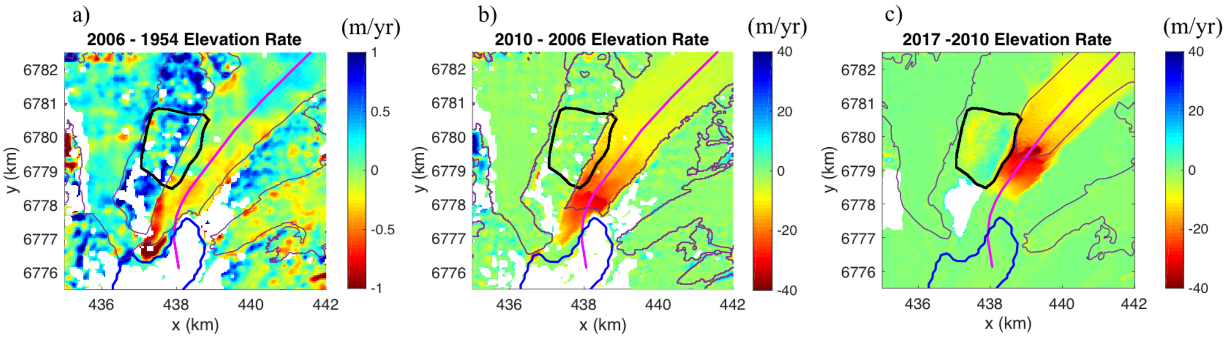

Fig. S3. Barry Glacier Surface Elevation Rate. (a) Rate of elevation change between 1954 and 2006 (data from *Berthier et al.,* 2010). The blue line is the GSHHS coastline, and the thin black lines are the glacier outlines from Randolph Glacier Inventory (data acquired on July 16, 2007). The thick black line and magenta line denote the landslide outline and the glacier centerline, respectively. (b) Rate of elevation change between 2006 and July 10, 2010 (IFSAR). The glacier outlines (thin lines) were acquired on August 3, 2009 (RGI). (c) Rate of elevation change between July 10, 2010 (IFSAR) and February 3, 2017 (ArcticDEM). The glacier outlines (thin lines) were acquired in July 2018 (RGI). All pairs of DEMs are coregistered with each other.

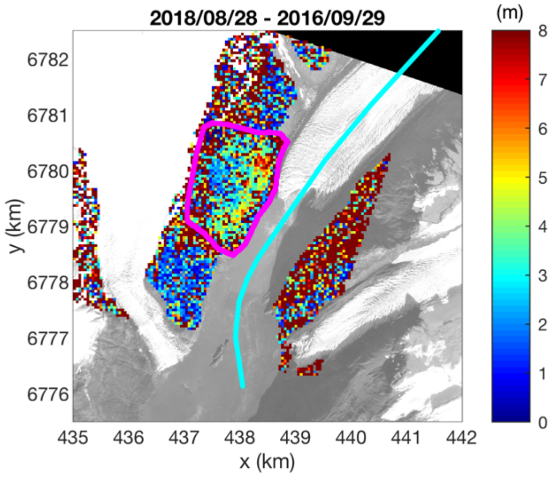

Fig. S4. The horizontal displacement from Planet images between 28 August 2018 and 29 September 2016. x and y are UTM zone 6N coordinates. Similar to Fig. 3, the data over glaciers and water areas are masked, and the translational offsets between images have been applied. The grayscale background is the 29 September 2016 Planet image, the magenta line is the landslide outline, and the cyan line is the Barry Glacier centerline. The standard deviations of the displacement over control points are 10 m (east) and 12 m (north). The median displacement over the landslide area is 3.3 m for u_x and -1.7 m for u_y., yielding a horizontal motion rate of 1.9 m/yr.

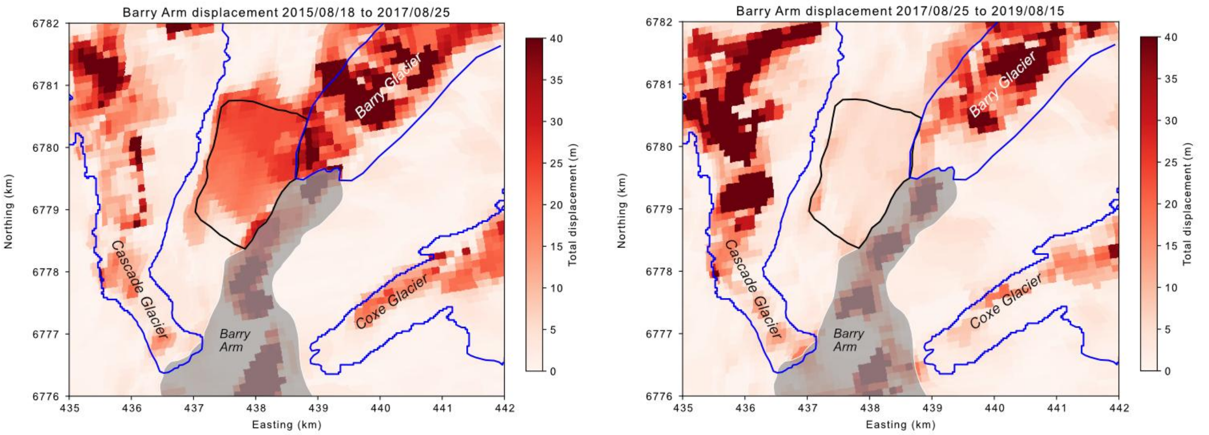

Fig. S5: Horizontal displacement magnitude of the Barry Arm landslide (black outline) between 18 August 2015 and 25 August 2017 (left) and 25 August 2017 and 15 August 2019 (right) mapped using offset correlation in Sentinel-1 amplitude images. Mean displacement on the landslide between 2015 and 2017 amounts to 19.8 m or ~10 m/y (mean absolute error = 1.9 m). Mean displacement between 2017 and 2019 is 5.12 m (mean absolute error = 1.3 m). High displacement rates in the Barry Arm fjord are likely due to icebergs and high sediment load. Axes are UTM Zone 6. Cascade and Coxe Glacier outlines are from the Randolph Glacier Inventory (RGI Consortium, 2017), the landslide outline, Barry Arm and Barry Glacier outlines were mapped manually.

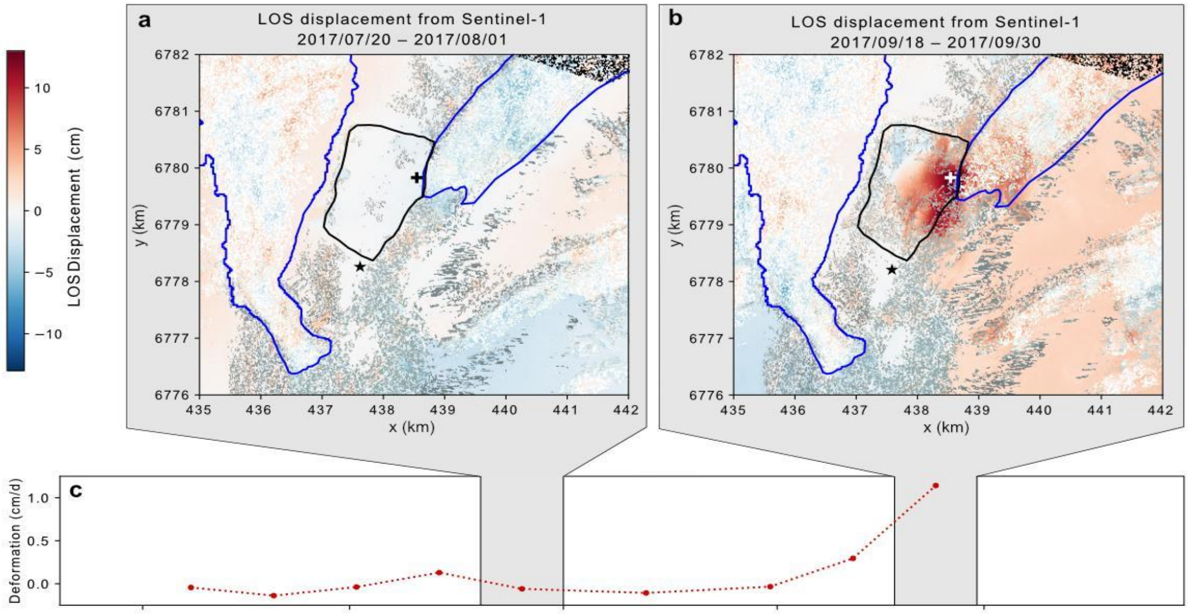

Fig. S6. Line of sight displacement from Sentinel 1 between 20 July and 1 August 2017 (**a**) and 18 and 30 September 2017 (**b**). Positive values denote ground motion toward the satellite (upward). Bottom panel (**c**): Daily displacement near the current glacier terminus (position marked with + in panels (a) and (b), values are averages of 100 x 100m area). The gray areas denote the time spanned by the corresponding interferograms. In panels (a) and (b), all pixels with an interferometric coherence of less than 0.3 have been masked. The black star indicates the stable reference area, the black line is the landslide outline, and the blue lines denote Cascade and Barry Glaciers. Copernicus Sentinel data (2017) retrieved from ASF DAAC (Alaska Satellite Facility Distributed Active Archive Center) (last accessed in May 2020), processed by ESA (European Space Agency).

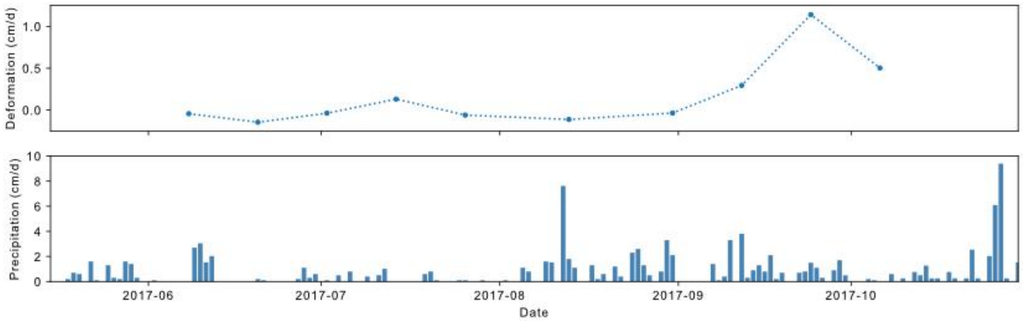

Fig. S7: Line of sight displacement of Barry Arm landslide during summer of 2017 and precipitation (from Esther Island Snowtel Station; https://wcc.sc.egov.usda.gov/nwcc/site?sitenum=1071). Upper panel is the same as Fig. S6(c), which is included here for easy comparison with precipitation. Positive values denote ground motion toward the satellite (upward).

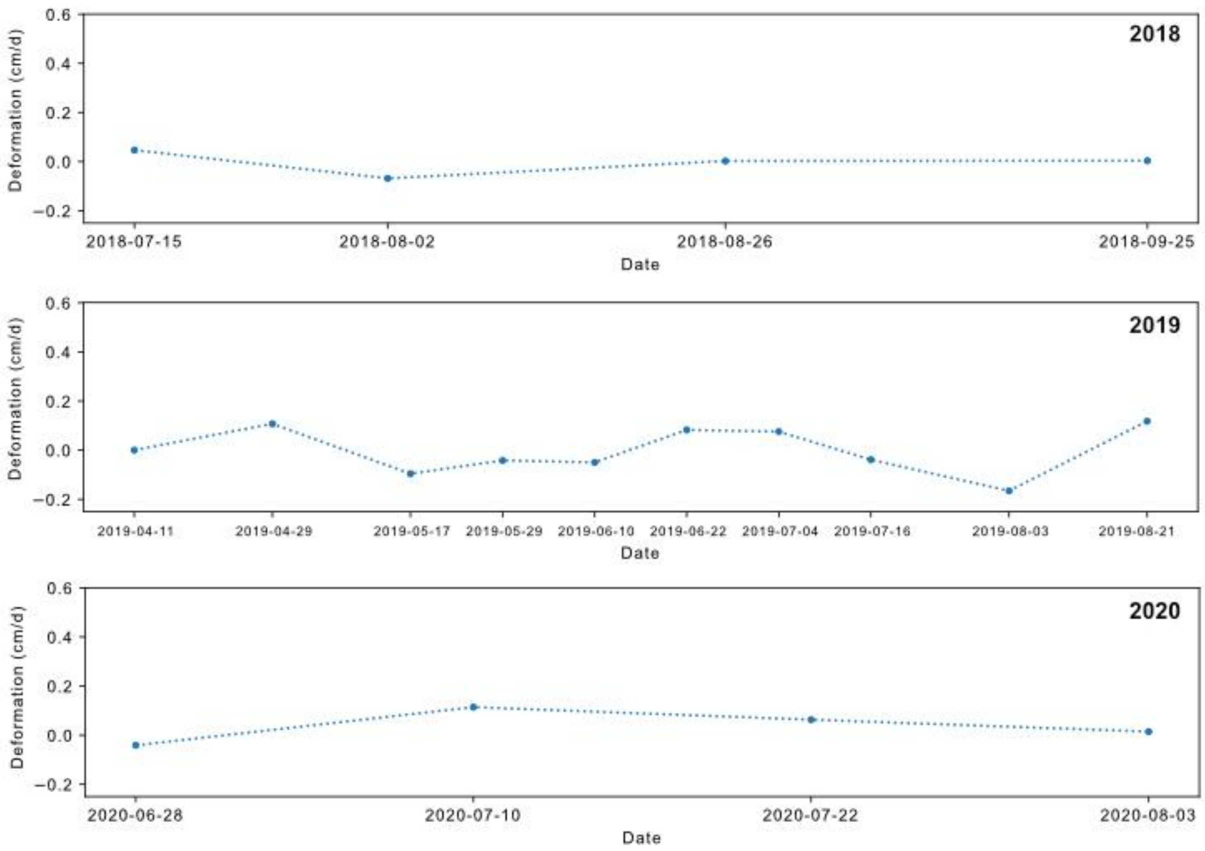

Fig. S8: InSAR time series derived from Sentinel-1A/B data during the summers of 2018, 2019, 2020. Velocities represent the mean value of a 100x100m pixel (see Fig. S6 for location).

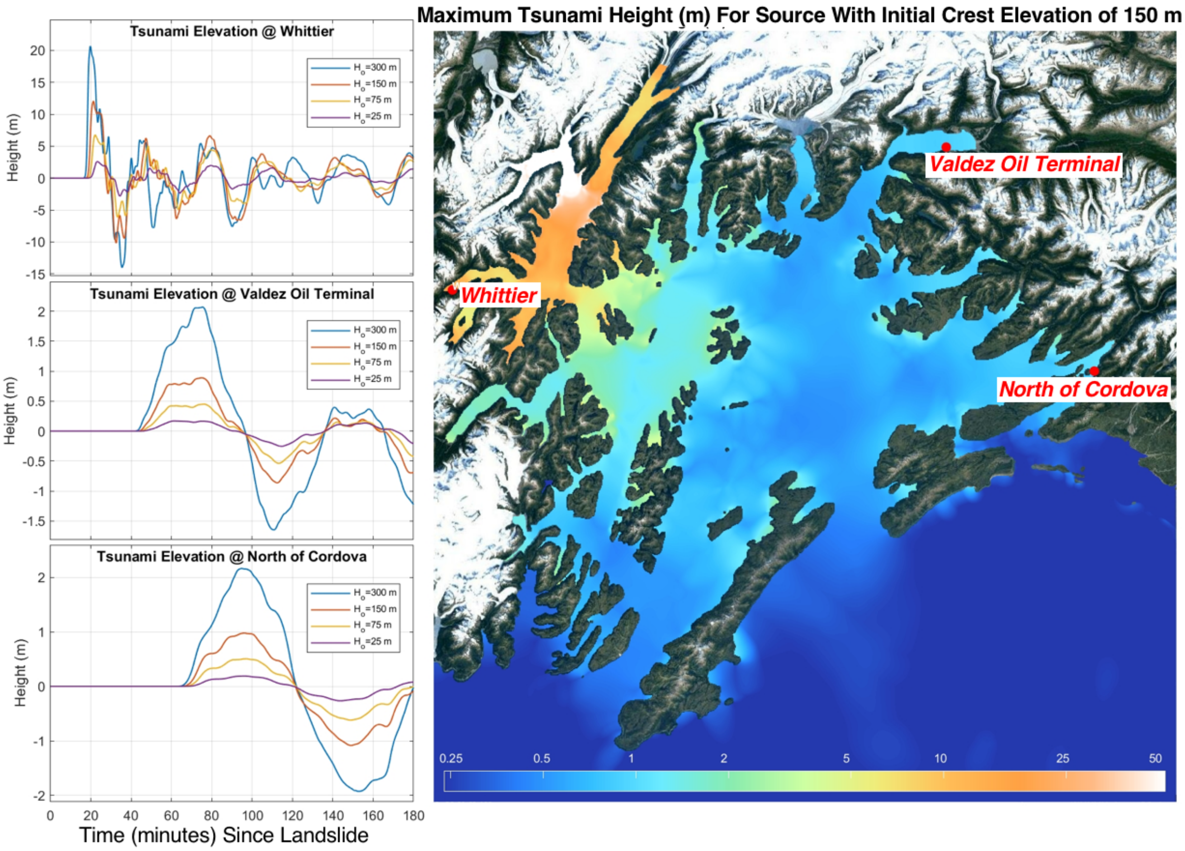

Fig. S9. Summary of numerically predicted tsunami elevations. Left panel: Time series of tsunami elevation with respect to sea level at Whittier (top), Valdez (middle), and Cordova (bottom). For each location, time series are provided for four different initial conditions, with initial crest elevations of 25, 75, 150, and 300 m. Right panel: Maximum tsunami elevation (m) throughout Prince William Sound, for the initial tsunami condition with 150 m crest elevation. Note that the three locations with time series data are shown by the red dots. Imagery © 2020 Maxar Technologies, retrieved from Google Earth 2020.

Table S1. List of optical satellite images used.

Acquisition Date (YYYYMMDD)	Satellite	Filename
20000624	ASTER	AST_L1T_00306242000213418_20150409231130_50053_V.tif
20020310	ASTER	AST_L1T_00303102002212025_20150421191215_55708_V.tif
20021112	ASTER	AST_L1T_00311122002212557_20150426081732_12382_V.tif
20050809	ASTER	AST_L1T_00308092005211824_20150510164810_106528_V.tif
20060429	ASTER	AST_L1T_00304292006212432_20150514043317_89008_V.tif
20070227	ASTER	AST_L1T_00302272007212526_20150518111423_86222_V.tif
20080621	ASTER	AST_L1T_00306212008212530_20150524111252_2363_V.tif
20100526	ASTER	AST_L1T_00305262010212510_20150601144430_84810_V.tif
20110927	ASTER	AST_L1T_00309272011211825_20150607194411_118314_V.tif
20140926	ASTER	AST_L1T_00309262014212529_20150623101558_5813_V.tif
20150828	ASTER	AST_L1T_00308282015212603_20150829164504_8158_V.tif
20150830	ASTER	AST_L1T_00308302015211349_20150831141954_19148_V.tif
20150922	ASTER	AST_L1T_00309222015211930_20150923235559_4257_V.tif
20160720	ASTER	AST_L1T_00307202016213213_20160721070944_22855_V.tif
20170709	ASTER	AST_L1T_00307092017211916_20170710102105_13989_V.tif
20170723	ASTER	AST_L1T_00307232017213137_20170724111640_5822_V.tif
20190708	ASTER	AST_L1T_00307082019211255_20190709110356_30330_V.tif
20191003	ASTER	AST_L1T_00310032019211850_20191006203156_15295_V.tif
19990731	LANDSAT 7	LE07_L1TP_068017_19990731_20161003_01_T1_B8.TIF
20000412	LANDSAT 7	LE07_L1TP_068017_20000412_20161002_01_T1_B8.TIF
20010915	LANDSAT 7	LE07_L1TP_067017_20010915_20160929_01_T1_B8.TIF
20020121	LANDSAT 7	LE07_L1TP_067017_20020121_20160929_01_T1_B8.TIF
20020402	LANDSAT 7	LE07_L1TP_068017_20020402_20160928_01_T1_B8.TIF
20020801	LANDSAT 7	LE07_L1TP_067017_20020801_20160928_01_T1_B8.TIF
20020902	LANDSAT 7	LE07_L1TP_067017_20020902_20160928_01_T1_B8.TIF
20130326	LANDSAT 8	LC08_L1TP_067017_20130326_20170310_01_T1_B8.TIF
20130915	LANDSAT 8	LC08_L1TP_068017_20130915_20170308_01_T1_B8.TIF
20140319	LANDSAT 8	LC08_L1TP_067017_20140319_20170307_01_T1_B8.TIF
20150202	LANDSAT 8	LC08_L1TP_067017_20150202_20180202_01_T1_B8.TIF
20150617	LANDSAT 8	LC08_L1TP_068017_20150617_20170226_01_T1_B8.TIF
20150804	LANDSAT 8	LC08_L1TP_068017_20150804_20170226_01_T1_B8.TIF
20150813	LANDSAT 8	LC08_L1TP_067017_20150813_20170226_01_T1_B8.TIF
20160527	LANDSAT 8	LC08_L1TP_067017_20160527_20180202_01_T1_B8.TIF
20160831	LANDSAT 8	LC08_L1TP_067017_20160831_20170222_01_T1_B8.TIF
20161025	LANDSAT 8	LC08_L1TP_068017_20161025_20170219_01_T1_B8.TIF
20170302	LANDSAT 8	LC08_L1TP_068017_20170302_20170316_01_T1_B8.TIF
20170311	LANDSAT 8	LC08_L1TP_067017_20170311_20170317_01_T1_B8.TIF
20170530	LANDSAT 8	LC08_L1TP_067017_20170530_20170615_01_T1_B8.TIF
20170615	LANDSAT 8	LC08_L1TP_067017_20170615_20170629_01_T1_B8.TIF
20171113	LANDSAT 8	LC08_L1TP_068017_20171113_20171122_01_T1_B8.TIF
20180109	LANDSAT 8	LC08_L1TP_067017_20180109_20180119_01_T1_B8.TIF
20180201	LANDSAT 8	LC08_L1TP_068017_20180201_20180220_01_T1_B8.TIF
20180321	LANDSAT 8	LC08_L1TP_068017_20180321_20180403_01_T1_B8.TIF
20180602	LANDSAT 8	LC08_L1TP_067017_20180602_20180615_01_T1_B8.TIF

20180929	LANDSAT 8	LC08_L1TP_068017_20180929_20181010_01_T1_B8.TIF
20181008	LANDSAT 8	LC08_L1TP_067017_20181008_20181029_01_T1_B8.TIF
20181031	LANDSAT 8	LC08_L1TP_068017_20181031_20181115_01_T1_B8.TIF
20190119	LANDSAT 8	LC08_L1TP_068017_20190119_20190201_01_T1_B8.TIF
20190204	LANDSAT 8	LC08_L1TP_068017_20190204_20190221_01_T1_B8.TIF
20190621	LANDSAT 8	LC08_L1TP_067017_20190621_20190704_01_T1_B8.TIF
20190628	LANDSAT 8	LC08_L1TP_068017_20190628_20190706_01_T1_B8.TIF
20190707	LANDSAT 8	LC08_L1TP_067017_20190707_20190719_01_T1_B8.TIF
20190808	LANDSAT 8	LC08_L1TP_067017_20190808_20190820_01_T1_B8.TIF
20200115	LANDSAT 8	LC08_L1TP_067017_20200115_20200127_01_T2_B8.TIF
20200122	LANDSAT 8	LC08_L1TP_068017_20200122_20200128_01_T1_B8.TIF
20200310	LANDSAT 8	LC08_L1TP_068017_20200310_20200325_01_T1_B8.TIF
20020402	Ikonos	IK01_20020402213700_2002040221371390000010000699_po_945612_pan_0000000
20100427	WorldView-1	WV01_20100427213038_102001000DA0E100_10APR27213038-P1BS-052121841010_04_P007
20160929	PlanetScope	20160929_202103_0e0e_3B_Visual.tif
20180828	PlanetScope	20180828_204132_1044_3B_Visual.tif

*Shadings are used to separate different satellites.

Table S2: List of all available Sentinel-1 InSAR scenes

Platform	Acquisition Date	Image File
S1A	20150514	S1A_IW_SLC__1SSV_20150514T033545_20150514T033615_005912_0079DD_19B7.zip
S1A	20150607	S1A_IW_SLC__1SSV_20150607T033547_20150607T033617_006262_008337_7235.zip
S1A	20150701	S1A_IW_SLC__1SSV_20150701T033548_20150701T033618_006612_008D0F_D504.zip
S1A	20150725	S1A_IW_SLC__1SSV_20150725T033549_20150725T033619_006962_0096F3_C07F.zip
S1A	20150818	S1A_IW_SLC__1SSV_20150818T033550_20150818T033620_007312_00A08C_0FD7.zip
S1A	20150911	S1A_IW_SLC__1SSV_20150911T033551_20150911T033621_007662_00AA1B_DF65.zip
S1A	20151005	S1A_IW_SLC__1SSV_20151005T033552_20151005T033622_008012_00B36B_7619.zip
S1A	20160812	S1A_IW_SLC__1SDV_20160812T033554_20160812T033624_012562_013AE4_EDBD.zip
S1B	20170509	S1B_IW_SLC__1SDV_20170509T033512_20170509T033542_005516_009AA2_B77B.zip
S1B	20170521	S1B_IW_SLC__1SDV_20170521T033513_20170521T033543_005691_009F84_FC0D.zip
S1B	20170602	S1B_IW_SLC__1SDV_20170602T033513_20170602T033543_005866_00A48F_2543.zip
S1B	20170614	S1B_IW_SLC__1SDV_20170614T033514_20170614T033544_006041_00A9B4_FB26.zip
S1B	20170626	S1B_IW_SLC__1SDV_20170626T033515_20170626T033545_006216_00AECB_2891.zip
S1B	20170708	S1B_IW_SLC__1SDV_20170708T033515_20170708T033545_006391_00B3BE_8306.zip
S1B	20170720	S1B_IW_SLC__1SDV_20170720T033516_20170720T033546_006566_00B8BF_EC7A.zip
S1B	20170801	S1B_IW_SLC__1SDV_20170801T033517_20170801T033547_006741_00BDC4_44B2.zip
S1B	20170825	S1B_IW_SLC__1SDV_20170825T033518_20170825T033548_007091_00C7ED_4954.zip
S1B	20170906	S1B_IW_SLC__1SDV_20170906T033518_20170906T033548_007266_00CD02_C893.zip
S1B	20170918	S1B_IW_SLC__1SDV_20170918T033519_20170918T033549_007441_00D227_0E60.zip
S1B	20170930	S1B_IW_SLC__1SDV_20170930T033519_20170930T033549_007616_00D72D_182E.zip
S1B	20171012	S1B_IW_SLC__1SDV_20171012T033519_20171012T033549_007791_00DC29_EF50.zip
S1B	20180504	S1B_IW_SLC__1SDV_20180504T033518_20180504T033548_010766_013AC7_6EBE.zip
S1B	20180528	S1B_IW_SLC__1SDV_20180528T033519_20180528T033549_011116_014622_BBA9.zip
S1B	20180703	S1B_IW_SLC__1SDV_20180703T033522_20180703T033552_011641_015681_DCDD.zip
S1B	20180727	S1B_IW_SLC__1SDV_20180727T033523_20180727T033553_011991_016130_4601.zip
S1B	20180808	S1B_IW_SLC__1SDV_20180808T033524_20180808T033553_012166_016683_2A76.zip
S1B	20180913	S1B_IW_SLC__1SDV_20180913T033526_20180913T033555_012691_0176B9_6C07.zip
S1B	20181007	S1B_IW_SLC__1SDV_20181007T033526_20181007T033556_013041_018174_82E4.zip
S1B	20190405	S1B_IW_SLC__1SDV_20190405T033524_20190405T033553_015666_01D625_3A3F.zip
S1B	20190417	S1B_IW_SLC__1SDV_20190417T033524_20190417T033554_015841_01DBF5_24AE.zip
S1B	20190511	S1B_IW_SLC__1SDV_20190511T033525_20190511T033555_016191_01E77C_9B20.zip
S1B	20190523	S1B_IW_SLC__1SDV_20190523T033526_20190523T033555_016366_01ECE6_D8C9.zip
S1B	20190604	S1B_IW_SLC__1SDV_20190604T033526_20190604T033556_016541_01F234_959F.zip
S1B	20190616	S1B_IW_SLC__1SDV_20190616T033527_20190616T033557_016716_01F763_9C54.zip
S1B	20190628	S1B_IW_SLC__1SDV_20190628T033528_20190628T033557_016891_01FC8C_82BD.zip
S1B	20190710	S1B_IW_SLC__1SDV_20190710T033528_20190710T033558_017066_0201BD_C502.zip
S1B	20190722	S1B_IW_SLC__1SDV_20190722T033529_20190722T033559_017241_0206D4_1940.zip
S1B	20190815	S1B_IW_SLC__1SDV_20190815T033531_20190815T033600_017591_021170_2DB9.zip
S1B	20190827	S1B_IW_SLC__1SDV_20190827T033531_20190827T033601_017766_0216EA_6B29.zip

S1B	20190920	S1B_IW_SLC__1SDV_20190920T033532_20190920T033602_018116_0221C7_55A3.zip
S1B	20191002	S1B_IW_SLC__1SDV_20191002T033533_20191002T033602_018291_022735_A731.zip
S1B	20200622	S1B_IW_SLC__1SDV_20200622T033534_20200622T033604_022141_02A05A_0818.zip
S1B	20200704	S1B_IW_SLC__1SDV_20200704T033534_20200704T033604_022316_02A5B2_787A.zip
S1B	20200716	S1B_IW_SLC__1SDV_20200716T033535_20200716T033605_022491_02AAFE_6859.zip
S1B	20200728	S1B_IW_SLC__1SDV_20200728T033536_20200728T033606_022666_02B054_C46C.zip
S1B	20200809	S1B_IW_SLC__1SDV_20200809T033537_20200809T033606_022841_02B5B0_ECB7.zip

*Shadings are used to separate different years of images.
